# Effects of Humic Substances and Mycorrhizal Fungi on Drought-Stressed Cactus: Focus on Growth, Physiology, and Biochemistry

**DOI:** 10.3390/plants12244156

**Published:** 2023-12-14

**Authors:** Soufiane Lahbouki, Ana Luísa Fernando, Carolina Rodrigues, Raja Ben-Laouane, Mohamed Ait-El-Mokhtar, Abdelkader Outzourhit, Abdelilah Meddich

**Affiliations:** 1 Center of Agrobiotechnology and Bioengineering, Research Unit Labelled CNRST (Centre AgroBiotech-URL-CNRST-05), “Physiology of Abiotic Stresses” Team, Cadi Ayyad University, Marrakech 40000, Morocco; benlaouaneraja@gmail.com; 2 Laboratory of Agro-Food, Biotechnologies and Valorization of Plant Bioresources (AGROBIOVAL), Department of Biology, Faculty of Science Semlalia, Cadi Ayyad University, Marrakesh 40000, Morocco; mohamed.aitelmokhtar@gmail.com; 3 MEtRICs/CubicB, Departamento de Química, NOVA School of Science and Technology, FCT NOVA, Universidade Nova de Lisboa, Campus de Caparica, 2829-516 Caparica, Portugal; ala@fct.unl.pt (A.L.F.); cpe.rodrigues@campus.fct.unl.pt (C.R.); 4 Laboratory of Nanomaterials for Energy and Environment Physics Department, Faculty of Sciences Semlalia, Cadi Ayyad University, P.O. Box 2390, Marrakech 40000, Morocco; aoutzour@uca.ac.ma; 5 Laboratory of Biochemistry, Environment & Agri-Food URAC 36, Department of Biology, Faculty of Science and Techniques—Mohammedia, Hassan II University of Casablanca, Mohammedia 20000, Morocco

**Keywords:** cactus, humic substances, arbuscular mycorrhizal fungi, drought stress, amino acids, ascorbic acid, mineral nutrition

## Abstract

Utilizing water resources rationally has become critical due to the expected increase in water scarcity. Cacti are capable of surviving with minimal water requirements and in poor soils. Despite being highly drought-resistant, cacti still faces limitations in realizing its full potential under drought-stress conditions. To this end, we investigated the interactive effect of humic substances (Hs) and arbuscular mycorrhizal fungi (AMF) on cactus plants under drought stress. In the study, a cactus pot experiment had three irrigation levels (W1: no irrigation, W2: 15% of field capacity, and W3: 30% of field capacity) and two biostimulants (Hs soil amendment and AMF inoculation), applied alone or combined. The findings show that the W1 and W2 regimes affected cactus performance. However, Hs and/or AMF significantly improved growth. Our results revealed that drought increased the generation of reactive oxygen species. However, Hs and/or AMF application improved nutrient uptake and increased anthocyanin content and free amino acids. Furthermore, the soil’s organic matter, phosphorus, nitrogen, and potassium contents were improved by the application of these biostimulants. Altogether, using Hs alone or in combination with AMF can be an effective and sustainable approach to enhance the tolerance of cactus plants to drought conditions, while also improving the soil quality.

## 1. Introduction

The world’s food security is being affected by a combination of interconnected and complex challenges [1,2]. Population increase and climate change are the two key problems [1,3]. The effects of climate change can be particularly severe in arid and semi-arid areas [4].

*Opuntia* is a type of succulent plant that has adapted to save water, allowing it to survive dry conditions [5,6]. They are able to absorb carbon dioxide at night thanks to a unique photosynthetic pathway called crassulacean acid metabolism (CAM) [7,8]. *Opuntia* is a valuable crop for food security and climate change adaptation in arid and semi-arid regions, where limited water availability limits the growth of other crops [9,10]. Although *Opuntia* can adapt to drought, it is also not completely immune to its negative effects. Numerous previous studies have shown that severe and prolonged dehydration can have a significant negative impact on their growth, biomass composition, and physiology, including biochemistry [9,11,12,13]. To solve this problem, the application of organic amendments, such as humic substances, and symbiotic microorganisms can be a solution for sustainable agriculture and to minimize the effects on yields and biomass composition changes of *Opuntia*.

Humic substances (Hs), which include both humic acid (Ha) and fulvic acids (Fa), are frequently utilized in agriculture as biostimulants to increase crop productivity and yield [14]. They can improve the physical and chemical properties of the soil [15]. Therefore, the compounds of Hs create stable aggregates with soil particles and Hs [16,17]. This connection could improve the capacity of retaining water in soil [15]. In addition, it has been shown that the application of Hs has a strong hormonal activity that improves plant growth [18,19]. It was also effective in improving the metabolic processes of plants, which are involved in the synthesis of nucleic acids and amino acids, and the antioxidant metabolism and mineral nutrition of plants, which can help mitigate the effects of abiotic stress, especially drought [15,20,21].

Arbuscular mycorrhizal fungi (AMF) and terrestrial plants create one of the most common symbioses in terrestrial plant ecosystems [22]. This symbiosis improves the plant’s buffering capacity against drought, due to the creation of arbuscular structures within the roots of the host plant providing a pathway for water transport between the fungus and its host, as well as facilitating nutrient uptake from the soil to the plant [23,24,25]. In order to obtain water and nutrients that are outside of the plant’s root system, AMF stretch their hyphae (fungal filaments) into the soil around them [26,27]. Additionally, they have the potential to synthesize glomalin, a glycoprotein that aids in stabilizing soil aggregates and enhancing soil water-holding capacity [28,29,30]. The plant–AMF symbiosis’s impact on general physiological and biochemical processes such as increasing the expression of genes involved in water-use efficiency, osmotic regulation, enzymatic activities, photosynthesis, respiration, and plant metabolism [31,32,33,34,35]. This may lead to enhanced plant drought resilience and improved plant growth and productivity.

Based on a previous study by Lahbouki et al. [13], it was demonstrated that cactus plants exhibit vulnerability to a 15%-drought-condition drought after 4 months of its imposition. Added to this, the application of vermicompost with AMF can reduce its effect on the physiochemical parameters of cactus cladodes. In the present study, we tested two main hypotheses: (1) We suggested decreasing the rate of water stress and seeing the response of the cactus to no watering for 4 months. (2) We tested whether the Hs extracted from vermicompost applied alone or in combination with AMF can serve as a causal factor and aid in mitigating the impacts of drought by enhancing cactus drought tolerance. The aim of the combined use of humic substances and mycorrhizal fungi is to improve soil structure and the ability of mycorrhizal fungi to efficiently absorb nutrients for plant roots and thereby enhance the cactus’s drought tolerance [16,23]. As far as we know, this study is the first of its kind to show the effect of Hs and AMF applied separately or in combination on drought-stressed soil and the cactus drought response, particularly on the cladode productivity, anthocyanin content, free amino acids, ascorbic acid, and nutrient uptake of cladodes.

## 2. Results

### 2.1. Soil Analysis

The data presented in Table 1 suggest that the soil characteristics were affected by the interactions between drought, Hs, and AMF. Despite the applied conditions, all soil characteristics were found to be improved after the harvest compared to their initial state. The study found that amending the soil under W3 conditions resulted in a significant increase in the levels of total organic carbon (TOC), available phosphorus (AP), and nitrogen (N). However, electric conductivity (EC) values decreased in all the treated plants, regardless of the water regime. Under W1 conditions, the EC values were significantly increased compared to W3. Additionally, the pH, N, and AP content showed a significant increase in response to treatment with Hs, AMF, and Hs+AMF under the same conditions. Among the treatments, Hs+AMF resulted in the highest increase in K, Ca, and Mg (27, 0.93, and 16%, respectively) compared to the control W3 plants.

### 2.2. Root Colonization and Plant Growth

No root colonization was found in untreated and Hs-treated plants (Table 2). The exposure of cacti to drought in both treatments (W1 and W2) decreased the frequency of mycorrhization in inoculated plants. Under W1, the F and M values of AMF of the plants inoculated with the AMF alone or in combination with Hs were decreased by 56 and 63% for AMF frequency (F) and 80 and 78% for AMF intensity (M), respectively, compared to the control. Likewise, the application of Hs in the inoculated plants showed no significant difference in these two parameters compared to the plants inoculated only with AMF.

Growth parameters of cactus cladodes were enhanced in the W1 condition by all applied treatments. Hs, AMF, and Hs+AMF benefited the cladode area (58, 8, and 65%, respectively), cladode dry weight (Cdw) (38, 7, and 37%, respectively), root length (Rl) (64, 38 and 72%, respectively), and root dry weight (Rdw) (55, 16, and 50%, respectively) compared to the control plants.

**Table 1:** Effects of humic substances (Hs) and/or arbuscular mycorrhizal fungi (AMF) on soil physicochemical parameters before and after the experiment under various water regimes.

	Before Experiment	After Experiment											
Treatments		Without Irrigation				15% F.c				30% F.c			
		T	Hs	AMF	Hs+AMF	T	Hs	AMF	Hs+AMF	T	Hs	AMF	Hs+AMF
Ec mS (cm^−1^)	0.17 ± 0.03 ^cd^	0.29 ± 0.02 ^a^	0.21 ± 0.05 ^bc^	0.26 ± 0.06 ^ab^	0.21 ± 0.04 ^bc^	0.25 ± 0.04 ^ab^	0.20 ± 0.03 ^bc^	0.22 ± 0.02 ^b^	0.19 ± 0.02 ^c^	0.16 ± 0.04 ^cd^	0.13 ± 0.02 ^d^	0.14 ± 0.03 ^cd^	0.15 ± 0.03 ^cd^
Ph	8.47 ± 0.3 ^ef^	8.59 ± 0.2 ^cd^	8.69 ± 0.3 ^ab^	8.61 ± 0.2 ^c^	8.71 ± 0.3 ^a^	8.62 ± 0.4 ^bc^	8.71 ± 0.2 ^a^	8.62 ± 0.2 ^c^	8.73 ± 0.4 ^a^	8.51 ± 0.1 ^e^	8.54 ± 0.2 ^de^	8.5 ± 0.5 ^e^	8.57 ± 0.2 ^cd^
TOC (%)	1.00 ± 0.08 ^e^	0.84 ± 0.05 ^g^	0.98 ± 0.04 ^e^	0.92 ± 0.08 ^f^	1.06 ± 0.04 ^d^	0.87 ± 0.02 ^g^	1.14 ± 0.04 ^cd^	1.04 ± 0.05 ^de^	1.19 ± 0.04 ^bc^	1.10 ± 0.08 ^d^	1.21 ± 0.11 ^ab^	1.18 ± 0.09 ^bc^	1.27 ± 0.05 ^a^
AP (mg/Kg)	12.00 ± 0.82 ^d^	11.75 ± 0.32 ^e^	12.28 ± 0.87 ^cd^	11.92 ± 0.32 ^e^	12.36 ± 0.16 ^c^	12.00 ± 0.39 ^d^	12.58 ± 0.19 ^c^	12.06 ± 0.24 ^d^	12.36 ± 0.39 ^c^	12.04 ± 0.74 ^d^	13.23 ± 0.94 ^b^	14.11 ± 0.58 ^a^	14.36 ± 0.54 ^a^
N (mg/Kg)	314.00 ± 28.36 ^de^	310.25 ± 17.69 ^e^	319.25 ± 29.48 ^d^	314.75 ± 18.44 ^de^	322.19 ± 27.39 ^c^	310.45 ± 30.14 ^e^	326.36 ± 25.69 ^b^	314.75 ± 27.57 ^de^	322.19 ± 20.18 ^c^	314.23 ± 25.47 ^de^	341.36 ± 30.17 ^ab^	333.14 ± 19.63 ^b^	347.00 ± 21.58 ^a^
K (mg/Kg)	1020.21 ± 40.28 ^i^	1238.48 ± 38.19 ^g^	1515.36 ± 30.47 ^c^	1347.69 ± 41.28 ^f^	1568.40 ± 44.39 ^b^	1180.41 ± 30.20 ^h^	1586.39 ± 41.46 ^b^	1450.36 ± 32.42 ^d^	1620.32 ± 33.69 ^a^	1148.28 ± 25.85 ^i^	1359.23 ± 42.76 ^ef^	1122.74 ± 34.22 ^i^	1379.11 ± 29.70 ^e^
Ca mg/Kg)	3.20 ± 0.05 ^cd^	3.22 ± 0.08 ^cd^	3.18 ± 0.04 ^d^	3.22 ± 0.05 ^cd^	3.25 ± 0.05 ^bc^	3.21 ± 0.07 ^cd^	3.27 ± 0.03 ^c^	3.25 ± 0.02 ^bc^	3.27 ± 0.00 ^bc^	3.21 ± 0.03 ^cd^	3.31 ± 0.01 ^ab^	3.27 ± 0.02 ^c^	3.35 ± 0.04 ^a^
Mg (mg/Kg)	332.40 ± 14.36 ^c^	330.78 ± 20.52 ^c^	378.00 ± 8.69 ^b^	359.40 ± 30.32 ^bc^	382.11 ± 18.69 ^ab^	330.57 ± 20.11 ^c^	395.15 ± 15.39 ^a^	352.04 ± 20.39 ^c^	396.04 ± 20.38 ^a^	332.43 ± 21.48 ^c^	380.52 ± 20.10 ^ab^	343.67 ± 34.68 ^c^	394.02 ± 28.69 ^a^

T: control plants; Hs: plants amended with humic substances; AMF: plants inoculated with AMF; Hs+AMF: plants amended and inoculated with Hs and AMF; F.c: field capacity; EC: electrical conductivity; TOC: total organic carbon; N: nitrogen; AP: available phosphorus; K: potassium; Ca: calcium; Mg: magnesium. The data presented are the mean (±standard error) of three replicates and different letters show significant differences at *p* ≤ 0.05.

**Table 2:** Effects of humic substances (Hs) and/or arbuscular mycorrhizal fungi (AMF) on growth and AMF colonization rate of *Opuntia ficus-indica* under various water regimes.

Treatments	Water Regime	F %	M %	Number of Cladodes/Plants	Surface Area (cm^2^)	Cladode Dry Weight (g)	Root Length (cm)	Root Dry Weight (g)
T	Without irrigation	0.00 ± 0.00 ^f^	0.00 ± 0.00 ^f^	1.17 ± 0.56 ^f^	86.92 ± 11.60 ^e^	3.90 ± 0.18 ^e^	0.53 ± 0.12 ^i^	1.32 ± 0.23 ^gh^
	15% F.c	0.00 ± 0.00 ^f^	0.00 ± 0.00 ^f^	1.67 ± 0.67 ^e^	99.70 ± 9.91 ^e^	3.75 ± 0.52 ^e^	0.64 ± 0.10 ^h^	1.27 ± 0.26 ^h^
	30% F.c	0.00 ± 0.00 ^f^	0.00 ± 0.00 ^f^	2.50 ± 0.50 ^bc^	174.83 ± 12.68 ^dc^	7.13 ± 0.10 ^b^	1.10 ± 0.11 ^de^	2.48 ± 0.25 ^c^
Hs	Without irrigation	0.00 ± 0.00 ^f^	0.00 ± 0.00 ^f^	1.33 ± 0.44 ^ef^	137.39 ± 6.17 ^b^	5.40 ± 0.17 ^dc^	0.87 ± 0.09 ^f^	2.05 ± 0.11 ^d^
	15% F.c	0.00 ± 0.00 ^f^	0.00 ± 0.00 ^f^	2.00 ± 0.33 ^cd^	153.62 ± 9.19 ^dc^	5.73 ± 0.13 ^c^	0.90 ± 0.04 ^e^	1.82 ± 0.28 ^ef^
	30% F.c	0.00 ± 0.00 ^f^	0.00 ± 0.00 ^f^	3.17 ± 0.89 ^a^	225.41 ± 4.97 ^a^	9.22 ± 0.22 ^a^	1.77 ± 0.19 ^b^	3.45 ± 0.27 ^a^
AMF	Without irrigation	51.11± 10.18 ^cd^	34.71 ± 2.64 ^d^	1.50 ± 0.50 ^ef^	93.75 ± 7.81 ^e^	4.19 ± 0.08 ^e^	0.73 ± 0.04 ^g^	1.53 ± 0.13 ^f^
	15% F.c	60.00 ± 6.66 ^c^	41.17 ± 1.56 ^c^	1.50 ± 0.50 ^ef^	133.80 ± 9.79 ^d^	4.60 ± 1.03 ^de^	0.84 ± 0.11 ^f^	1.62 ± 0.15 ^f^
	30% F.c	80.00 ± 6.66 ^a^	62.31 ± 3.13 ^a^	3.00 ± 0.67 ^ab^	200.78 ± 5.75 ^ab^	8.02 ± 0.14 ^b^	1.44 ± 0.11 ^c^	2.80 ± 0.14 ^b^
Hs+AMF	Without irrigation	42.22 ± 3.84 ^e^	27.88 ± 2.62 ^e^	1.50 ± 0.83 ^ef^	143.05 ± 9.59 ^d^	5.53 ± 0.14 ^dc^	0.91 ± 0.09 ^e^	1.98 ± 0.08 ^e^
	15% F.c	48.88 ± 10.18 ^de^	36.4 ± 1.66 ^d^	1.83 ± 0.56 ^de^	144.72 ± 7.08 ^d^	5.58 ± 0.11 ^dc^	0.99 ± 0.10 ^e^	2.02 ± 0.15 ^de^
	30% F.c	68.88 ± 3.48 ^b^	49.86 ± 6.66 ^b^	3.00 ± 0.67 ^ab^	221.81 ± 12.50 ^a^	9.55 ± 0.09 ^a^	1.92 ± 0.10 ^a^	2.76 ± 0.25 ^bc^

T: control plants; Hs: plants amended with humic substances; AMF: plants inoculated with AMF; Hs+AMF: plants amended and inoculated with Hs and AMF; F.c: field capacity; F%: mycorrhizal frequency; Ma%: intensity. The data presented are the mean (±standard error) of three replicates and different letters show significant differences at *p* ≤ 0.05.

### 2.3. Stomatal Conductance and Malic Acid Content

**Figure 1:**
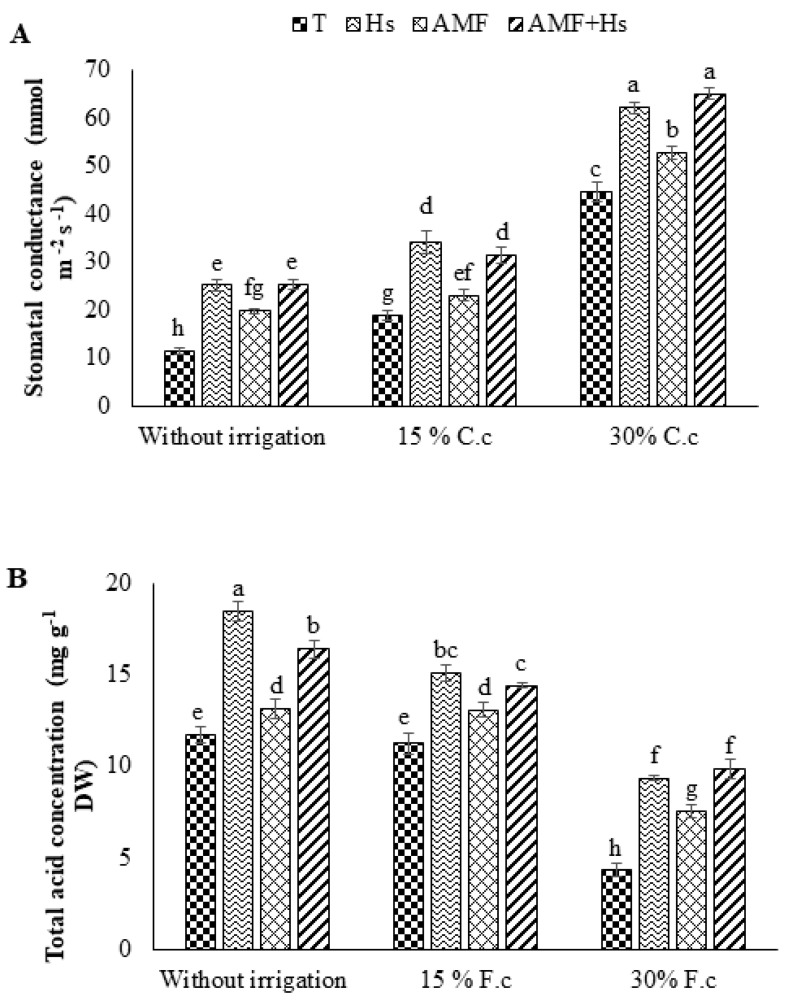
Influence of humic substances and/or arbuscular mycorrhizal fungi on (**A**) stomatal conductance and (**B**) total acid concentration at different drought levels in cactus plants. Different letters indicate significantly different values at *p* ≤ 0.05.

The statistical analysis showed that the response of stomatal conductance (gs) and malic acid content of cacti was highly significant between all applied water regimes (Figure 1). The absence of watering directly reduced the gs level in cactus plants. Conversely, under the same condition, the malic acid content was raised, all in comparison with the watered plants (W3). Moreover, Hs, AMF, and Hs+AMF plants enable increased gs (118, 72, and 120%, respectively) and malic acid content (58, 12, and 40%, respectively) compared to non-treated plants experimented in W1 conditions. Under W3, adding biostimulants had a substantial effect on increasing both parameters. Indeed, Hs were even more effective in improving them.

### 2.4. Oxidative Stress Indicators

**Figure 2:**
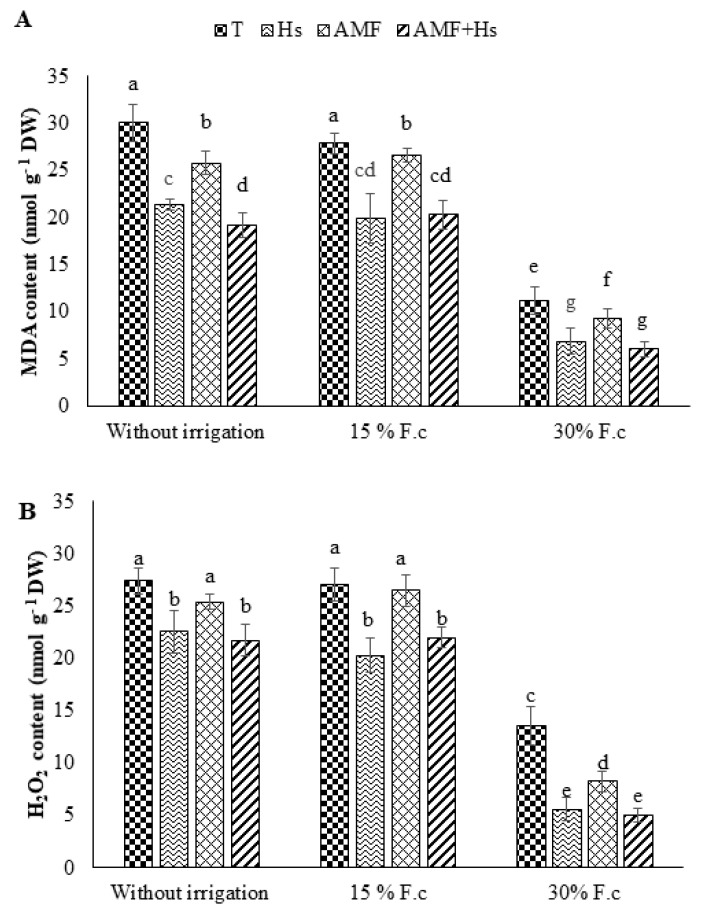
Influence of humic substances and/or arbuscular mycorrhizal fungi on (**A**) malondialdehyde (MDA) content and (**B**) hydrogen peroxide (H_2_O_2_) at different drought levels in cactus plants. Different letters indicate significantly different values at *p* ≤ 0.05.

Figure 2 depicts the effects of water regimes, Hs, AMF, and Hs+AMF separately and in combination on oxidative stress markers. The results showed that W1 increased malondialdehyde (MDA) and hydrogen peroxide (H_2_O_2_) levels by 63% and 50%, respectively, compared to the W3 plants. Nevertheless, biostimulant application attenuated the elevation of these stress markers and showed a decrease of 18 and 25% due to Hs, 7 and 2% due to AMF, and 21 and 19% due to Hs+AMF in MDA and H_2_O_2_, respectively, compared to W1 control plants.

### 2.5. Anthocyanin Content, Sugar, Free Amino Acids, and Ascorbic Acid

**Figure 3:**
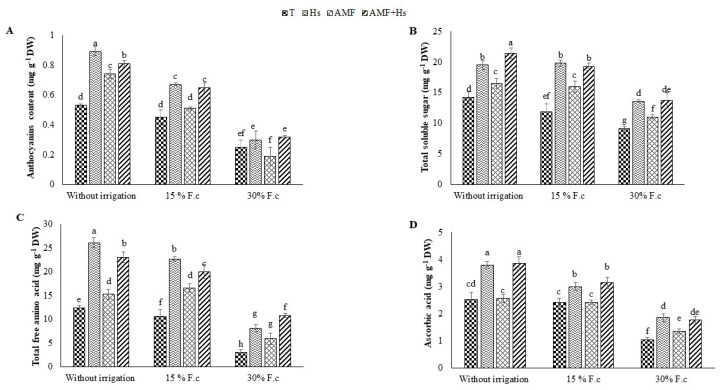
Influence of humic substances and/or arbuscular mycorrhizal fungi on (**A**) anthocyanin, (**B**) total soluble sugar, (**C**) free amino acid, and (**D**) ascorbic acid contents at different drought levels in cactus plants. Different letters indicate significantly different values at *p* ≤ 0.05.

According to Figure 3A, an increase in anthocyanin content was observed with increasing water stress levels in cactus cladodes. Under W1, when comparing all treatments to control plants, plants amended with Hs and/or inoculated with AMF increased the cladode anthocyanin content. This accumulation of anthocyanin reached higher values in plants amended with Hs followed by plants treated with Hs+AMF and inoculated with AMF by 6, 16, and 4%, respectively, than in control plants.

Figure 3B–D illustrate that the W1 and W2 treatments resulted in a significant increase in the levels of total soluble sugar (Tss), amino acids, and ascorbic acid. Moreover, the application of Hs, AMF, and Hs+AMF further increased these compounds by 37, 16, and 44%, respectively, for sugar; 111, 23, and, 86%, respectively, for amino acids; and 57, 34, and 59%, respectively, for ascorbic acid under W1, and by 66, 34, and 62%, respectively, for Tss; 114, 56, and 89%, respectively, for amino acid; and 50, 21, and 57%, respectively, for ascorbic acid under W2 compared to the control plants under the same regimes.

However, Figure 3D demonstrates that the single inoculation with AMF did not significantly increase the levels of ascorbic acid for both hydric levels compared to the control. Under W3, the application of these biostimulants resulted in an increase in all these compounds, including sugar, amino acids, and ascorbic acid.

### 2.6. Antioxidant Enzyme Activities

**Figure 4:**
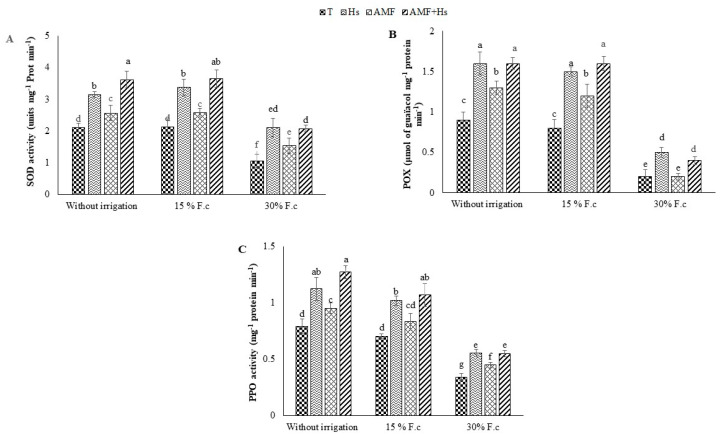
Influence of humic substances and/or arbuscular mycorrhizal fungi on (**A**) superoxide dismutase (SOD), (**B**) peroxidase (POX), and (**C**) polyphenol oxidase (PPO) activities at different drought levels in cactus plants. Different letters indicate significantly different values at *p* ≤ 0.05.

The data in Figure 4 show that drought application in both treatments—W1 and W2—caused an increase in superoxide dismutase (SOD), peroxidase (POX), and polyphenol oxidase (PPO) activities compared to W3 plants, while there were no significant differences in W1 and W2 plants. Furthermore, biostimulant application significantly increased antioxidant enzyme activities, independent of water treatments. Nevertheless, higher levels of antioxidant activities in treated plants were recorded in both W1 and W2. Application of Hs, AMF, and Hs+AMF increased SOD (49, 21, and 71% and 58, 21, and 71%, respectively), POX (77, 44, and 77% and 87, 50, and 100%, respectively), and PPO (42, 21, and 61% and 45, 18, and 52%, respectively) compared to the control of W3 plants.

### 2.7. Plant Nutrient Uptake Analysis

The changes in cladode nutrients content when subjected to different water and biostimulant treatments are shown in Table 3. Phosphorus (P) and N in cactus cladodes did not show significant variation regardless of whether the regimes were W1 or W2. However, the potassium (K) level decreased with the levels of water application. Meanwhile, Hs and/or AMF application increased the percentage of P, N, and K in the cactus cladodes in all water regimes. As an example, in W1 plants, the highest level of P increase was found in cactus cladodes inoculated with AMF. Regarding N and K content, the highest value was recorded in cladodes treated with Hs+AMF. It was 36 and 32%, respectively, compared to the control plants of the same regime.

### 2.8. Principal Component Analysis

**Figure 5:**
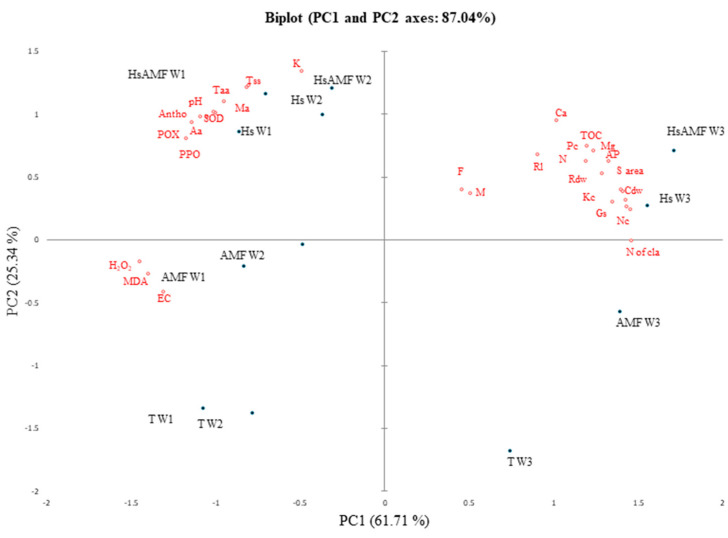
Principal component analysis (PCA) of cladodes grown under different water regimes (unirrigated plants (W1), plants irrigated at 15% of field capacity (W2), and plants irrigated at 30% of field capacity (W3)) and submitted to different biostimulant treatments after four months of drought-stress application. T: control treatment; Hs: plants amended with humic substances; AMF: plants inoculated with AMF consortium; Hs+AMF: plants amended with humic substances and inoculated with AMF consortium; EC: electric conductivity; Toc: total organic carbon; AP: available phosphorus content in soil; N: nitrogen content in soil; K: potassium content in soil; Ca: calcium content in soil; Mg: magnesium content in soil; F: root AMF colonization frequency; M: root AMF intensity; S area: surface area of cladodes; N of cla: number of new cladodes; Cdw: cladode dry weight; Rl: root length, Rdw: root dry weight; Gs: stomatal conductance; Ma: total acid concentration; MDA: malondialdehyde content; H_2_O_2_: hydrogen peroxide content; antho: anthocyanin content; Tss: total soluble sugar content; Taa: total free amino acid content; Aa: ascorbic acid content; SOD: superoxide dismutase content; POX: peroxidase content; PPO: polyphenol oxidase content; Nc: nitrogen content in cactus cladodes; Pc: phosphorus content in cactus cladodes; Kc: potassium content in cactus cladodes.

To better understand the relationship among biostimulants, drought, and the measured parameters, a PCA was carried out to identify the variables that have a strong impact on plant growth and stress tolerance (Figure 5). The PCA results showed that PC1 explained 61.71% and PC2 explained 25.34% of the total variance. The data showed that all biostimulants applied alone or in combination were separated from the untreated control. Biological treatments leading to higher biomass production and stress tolerance were on the right side of the PC1. Under W3, Hs alone or combined with AMF treatments was positively related to growth, AMF infection, stomatal conductance, and the nutrients phosphorus, nitrogen, and potassium in plants. In addition, the same treatments were closely related to the free amino acid, total soluble sugar, and antioxidant defense system under W1 and W2 and observed in the lower left panel of PC1, which could lead to a more complete understanding of the protective effects of biostimulants in cacti under drought stress.

## 3. Discussion

Drought and the increasing poverty of agricultural soils are a major concern, especially in arid and semi-arid regions, as they affect crop growth and lead to huge yield losses. This work ensured the ability of Hs and AMF applied separately or in combination to improve the ability of cacti to cope with drought stress by enhancing the soil characteristics and regulating cactus growth, physiology, and biochemical processes. Through soil analysis, it was found that drought had a significant negative impact on soil properties, namely, increased EC, decreased TOC, and decreased availability of AP, N, K, Ca, and Mg. The increase in EC caused by drought may cause soil structure degradation and limit water and nutrient availability to plants [36]. Our results are consistent with those observed in most previous studies [29,37]. The inclusion of both Hs and/or AMF had a direct positive influence on soil quality under drought. Humic substances enhanced the structure of soil by forming complexes with soil clay particles, which can help to stabilize the clay [38]. It can also enhance the nutrients in soil, especially AP, N, and Ca. This can be explained by the chelating capacity of Hs [16,39]. In addition, Hs can increase the proportion of exchangeable K^+^ in adsorbed K^+^, which increases K availability in soil [40]. In addition, AMF can solubilize the essential nutrients P and K in the soil, making them more available to plants [41]. Additionally, AMF can also aid in the biodegradation of soil organic matter and produce phytohormones, which can have positive effects on soil characteristics [42]. In this study, drought-affected root colonization in the cactus plant could be explained by the reduction in the development of AMF hyphae in the water-stressed soil [43]. Drought stress can decrease the photosynthetic capacity of host plants, which in turn can reduce the supply of carbohydrates available for both the plant and its associated microbes, including AMF [44].

The growth and development of mycorrhizal hyphae as well as the germination of AMF spores may be constrained by the decreased availability of carbohydrates [43,45]. Paradoxically, Hs application also decreased root colonization. This may be because Hs application can alter the soil pH and nutrient availability, especially P, which may reduce plants’ dependence on AMFs for phosphorus uptake and reduce the need for AMF colonization [46,47]. In the current study, drought caused a significant decrease in growth parameters. The decrease in growth parameters due to water-deficit conditions is consistent with current studies on lettuce by Ouhaddou et al. [37] and tomato by Lahbouki et al. [7]. The reduction in cactus growth under drought could be attributed to the reduction in photosynthetic capacity caused by excessive drought [13]. Furthermore, one of the cactus strategies to combat drought is reducing the cladode area to optimize transpiration [11]. The current findings demonstrated that Hs-AMF independently or in combination increased cactus growth in terms of cladode area and dry weight under drought conditions. Several other workers have reported similar effects of Hs-AMF enhancing plants’ growth for a range of other crops such as sugar beet [21], maize [15], and lettuce [37]. The beneficial effect of Hs on cactus growth under water-deficit conditions could be linked to the positive effect of Hs on improved fertility and structure of soil [15,16]. In this study, Hs led to an increase in TOC in soil. Hence, extensive studies have indicated that the increase in the soil TOC content improved soil ventilation and aggregation, which provide plants with more constant access to water through their effect on soil aggregates, which benefits crop growth [15,48]. Furthermore, their chelating character may allow the formation of more stable complexes with the metal ions necessary for plant growth such as K, Mg, and Ca, allowing a more efficient uptake by plant roots [39,49,50]. In addition, AMF can improve plant growth during drought by enhancing water and mineral uptake through fungal filaments, as well as promoting nutrient transport and phosphorus solubilization [51,52]. In addition, plant–AMF symbiosis promotes the growth of root hairs through its influence on auxin synthesis and accumulation [42]. It was also proved that Hs can accumulate auxins in the roots of plants [53].

In this investigation, the cactus plants were found to use multiple physiological and biochemical mechanisms to regulate their metabolism and modify their osmotic balance to combat the loss of water. Stomatal conductance was significantly reduced by water shortage. This reduction in gs is believed to be a response to water conservation mechanisms in the cladodes, as well as a decrease in the density and state of stomata [11,54,55] This reduction in gs can limit the uptake of CO_2_ for photosynthesis, and this in turn can limit the rate of photosynthesis and reduce net photosynthesis, which can lead to reduced growth [55]. Some previous studies have demonstrated that a reduction in the CO_2_ uptake in the cactus plants led to a reduction in malic acid consumption [56,57]. Therefore, an increase in malic acid accumulation occurred in response to the reduced availability of CO_2_. This finding is consistent with our results, which showed that drought causes an accumulation of malic acid in CAM plants.

In the presence of Hs-AMF, gs was increased under drought. This can be attributed to its role in improving the availability of nutrients in the soil, particularly N and AP, which are essential for photosynthetic rates [58,59]. Hence, Yamori et al. [60] demonstrated that an increase in photosynthetic rates can lead to higher gs. Furthermore, our study showed that Hs-AMF also increased K in plants. It is known that K plays a direct role in regulating gs by controlling the movement of ions and water in and out of the guard cells that surround the stomatal pore and by regulating the opening and closing of stomata [61]. Hence, increased plant K may increase gs. Hs-AMF may be able to increase the production of plant hormones, including abscisic acid and cytokinins [62,63]. These plant hormones are involved in the opening and closing of stomata as well as stomatal development [64].

Drought can cause damage to plants’ cellular structures, such as membrane lipids, proteins, and DNA, which is often caused by the accumulation of reactive oxygen species (ROS) [65]. Levels of MDA and H_2_O_2_ are widely measured to assess the production of ROS in plants [66]. Drought stress significantly increased MDA and H_2_O_2_ in cactus plants. In order to combat this increase, plants have evolved mechanisms to prevent the accumulation of ROS that can damage cells. These mechanisms include where they store certain types of osmoprotectants known as compatible solutes [67]. These solutes include Tss and ascorbic acid, which can help plants combat drought stress [13,67]. They are likely involved in maintaining water balance and turgor levels and supporting overall physiological traits [68,69]. Additionally, the accumulation of ascorbic acid and anthocyanin in plant tissues can act as antioxidants to scavenge ROS [70,71]. Our results suggest that the application of biostimulants increases the contents of these compounds under drought stress, indicating the role of these biostimulants in enhancing the ability of cacti to withstand drought stress, in accordance with previous studies [21,29]. The observed increase in compounds such as Tss, total free amino acid, ascorbic acid, and anthocyanin in plants may be due to the role played by Hs-AMF in increasing the proportion of P and N in plants [16,47,51]. Phosphorus and N are important macronutrients for plant growth and development and can directly or indirectly influence the biosynthesis of various compounds in plants, including total free amino acid, ascorbic acid, and anthocyanin [72,73]. At the same time, water-stress-tolerant plants can also respond to adapt to water stress by changing their cellular metabolism and activating numerous defensive mechanisms, such as the activation of antioxidant enzymes [21,29]. The first line of defense against oxidative damage is SOD, which converts highly reactive superoxide (O_2_^−^) into less dangerous H_2_O_2_ and molecular oxygen [74]. However, H_2_O_2_ is detoxified in plant cells by several enzymes, including POX and PPO, which change it into H_2_O and O_2_ [75]. Consequently, increased antioxidant metabolism can improve a plant’s ability to scavenge ROS [29]. Overall, the results revealed that combining soil amendment with Hs and/or native AMF led to higher antioxidant enzymes in plants. This result is in agreement with previous studies that have reported similar findings in corn [76] and sugar beet [21].

Overall, fulfilling our hypothesis, the results confirmed that the cacti grown in the greenhouse were able to survive for 4 months without water, yet their morphological and biochemical parameters were still highly affected. Furthermore, the application of Hs led to an improvement in the cacti’s ability to endure drought at both moderate and severe levels. This finding supports the assertion that Hs play a crucial role in enhancing the drought tolerance of cacti, regardless of whether they are applied alone or in combination with AMF.

## 4. Materials and Methods

### 4.1. Biological and Biostimulant Materials

Cactus cladodes used for planting were one-year-old cladodes of *Opuntia ficus-indica*, collected locally from the Rhamna region, Morocco (31°48′22.4″ N 7°58′36.6″ W). The used cladodes were left to dry for two weeks in the shade to allow the healing of the cut areas. In plastic pots, one cladode was planted in 2.5 kg of soil (previously sterilized at 200 °C for 3 h). The properties of the soil mixture used are as follows: it contained 16.00% clay, 3.00% coarse silt, 9.00% fine silt, 42.30% coarse sand, and 29.70% fine sand.

Humic substances were extracted from vermicompost according to the method of Stevenson [77], using a solution of NaOH (1:5) with shaking for 2 h and precipitation for 24 h with (3 N) H_2_SO_4_ to pH 2. Humic and fulvic acid purification was performed by adding a solution of oxalic acid and (6 N) sulfuric acid [77]. Humic substance products had 70.00% humic acid and 30.00% fulvic acid, pH 6.8, TOC: 0.94%.

The mycorrhizal consortium used in the present investigation contained spores and fragments of infested maize roots with 10 g of AMF per plant. The consortium consisted of 1034 spores/100 g soil in 22 species: *Acaulospora mellea*; *Acaulospora laevis*; *Acaulospora delicata*; *Acaulospora bireticulata*; *Acaulospora myriocarpa*; *Glomus* sp.; *Glomus microcarpum*; *Glomus macrocarpum*; *Glomus globiferum*; *Glomus rubiforme*; *Glomus multicaule*; *Glomus heterosporum*; *Rhizophagus intraradices*; *Rhizophagus aggregatus*; *Rhizophagus fasciculatus*; *Scutellospora nigra*; *Scutellospora heterogama*; *Gigaspora margarita*; *Claroideoglomus drummondii*; *Claroideoglomus etunicatum*; *Diversispora versiformis*; *Diversispora trimurales* [78].

### 4.2. Experimental Design

The experiment was arranged in pots in the greenhouse at the Faculty of Science Semlalia, Cadi Ayyad University, Marrakesh, Morocco, with a 16 h light (410 μmol photons s^−1^ m^−2^)/8 h dark photoperiod, with temperatures set at 25 °C during the light period and 21 °C during the dark period and a relative humidity of 40–60%. It was designed as a factorial experiment based on a randomized complete block design containing four groups with 18 replicates per group (resulting in a total of 72 pots). The groups were treated as follows: control T (no amendment), Hs (plants amended with humic substances at a rate of 0.1 mL/Kg according to Lermen et al. [79]), AMF (plants inoculated with 10 g/pot of AMF consortium according to Lahbouki et al. [13]) and Hs+AMF (plants supplemented with both Hs and AMF).

Each plant received a consistent volume of water twice a week (40 mL per watering). For each group and after four months of cultivation, the plants were divided into three subgroups: unirrigated plants (W1), plants irrigated at 15% of field capacity (W2), and plants irrigated at 30% of field capacity (W3). In total, 12 treatments were applied with 6 replicates for each. As stated by Meddich et al. [80], water stress was applied in these conditions for a duration of four months.

### 4.3. Soil Composition

Soil samples were collected both before the experiment with no applied treatments and after the experiment with treatments applied. These samples were air-dried and sieved to <1 mm for physicochemical property analysis. Electric conductivity and pH were measured in a 1/5 (*w*/*v*) diluted soil suspension using a conductivity meter HI-9033 (Hanna Instruments, Padua, Italy) and a pH meter (HI 9025). Total organic carbon was carried out according to Aubert [81]. Olsen and Sommers’s [82] method was used to calculate AP. N, K, Ca, and Mg concentrations were determined using ICP/OES Ultima Expert (Inductively Coupled Plasma/Optical Emission Spectrometry, iCAP 6500 Duo, Horiba Inc., Burlington, ON, Canada).

### 4.4. Estimation of Mycorrhizal Root Colonization and Plant Growth

Roots colonization by AMF was evaluated for each treatment using the method described by Phillips and Hayman [83]. Root samples were cleared with a 10% KOH solution and stained with 0.05% trypan blue in lactic acid. The stained segments (1 cm, 12 segments with 3 replicates for each sample) were observed under a Zeiss Axioskop 40 microscope at 40–100× magnification. Mycorrhizal frequency and M% of the stained roots were estimated using the method of Trouvelot and Kough [84].

The surface area of cactus cladodes was determined as described by Tiznado-Hernandez et al. [85]. Thereafter, the number of cladodes, Cdw, Rl, and Rdw were determined as detailed by Lahbouki et al. [13].

### 4.5. Stomatal Conductance and Malic acid Content

Stomatal conductance was measured between 2 and 4 a.m. due to the characteristic nocturnal stomatal opening of CAM plants with a porometer (CI-340, Handheld Photosynthesis System, W SA, CID-Science, Camas, WA, USA) by following the instructions in the user manual.

Adopting Ojeda-Pérez et al. [56], cactus samples were milled into 20 mL of 60% ethanol, boiled for 5 min, then titrated with 0.1 N NaOH to measure the total acid concentration (malic acid content).

### 4.6. Measurement of Oxidative Stress Indicators

Lipid peroxidation was performed on fresh cladode samples by measuring the malondialdehyde (MDA) content, which was quantified using thiobarbituric acid following Madhava and Sresty’s method [86]. The absorbance was recorded at 532 nm and the results were calculated by referring to a standard prepared MDA curve and were expressed as nmol MDA g^−1^ of dry weight (DW).

The method of Velikova et al. [87] was employed for the determination of H_2_O_2_ in the fresh cactus cladodes. The absorbance was recorded at 390 nm after 1 h of incubation in the dark. The activity of H_2_O_2_ was expressed in nmol H_2_O_2_ g^−1^ of DW. Lipid peroxidation was assessed utilizing H_2_O_2_ as a standard.

### 4.7. Anthocyanin Content

Analysis of anthocyanin content was performed by recording the absorbance at 657 nm and 530 nm according to the method of Baozhu et al. [88]. Following the protocol, 1 g of the sample was extracted with 600 μL of 1% (*v*/*v*) HCl-methanol. Subsequently, 600 μL of chloroform and 300 μL of distilled water were mixed with the extracts. The anthocyanin content was calculated using an established formula and was expressed as mg g^−1^ DW.

### 4.8. Estimation of Sugar, Free Amino Acids, and Ascorbic Acid

The total soluble sugar content of cactus cladodes was measured according to the procedures reported by Dubois et al. [89].

The total free amino acid content was determined with the ninhydrin method by recording the absorbance at 760 nm and expressing it as mg g^−1^ DW, as described by Lee and Takahashi [90]. The total free amino acid was calculated by referring to a standard prepared glycine curve.

Following the protocols developed by Adrian and Peiró [91], the ascorbic acid content was assessed titrimetrically. First, 1 g of fresh cladode was ground in 20 mL of distilled water (40%). Then, 1 mL of the sample was added to 1 mL of glacial acetic acid, then titrated with 2,6-dichlorophenol indophenol (2, 6-DCPIP) (0.025%) to a pink end point. The ascorbic acid content was determined using a comparison of the amount of 2,6-DCPIP reagent used in the titration with that used for known quantities of a standard solution containing 0.1% ascorbic acid.

### 4.9. Antioxidant Enzyme Activities

A 0.1 g sample of cactus cladode was shredded and ground in a mortar and pestle, and the resulting powder was homogenized in 5 mL of a solution containing 0.1 mol/L potassium phosphate buffer (pH 7.0), 0.1 g polyvinylpolypyrrolidone, and 0.1 mmol/L ethylenediaminetetraacetic acid (EDTA). It was then centrifuged at 18,000× *g* at 4 °C for 15 min. The eventual supernatant was used as an enzyme source for the determination of enzyme activities.

Nitro blue tetrazolium (NBT) was used for determining SOD using the method outlined by Beyer and Fridovich [92]. The reaction mixture was composed of sodium phosphate buffer (50 mM) with pH 7.6, EDTA (0.1 mM), sodium carbonate (50 mM), l-methionine (12 mM), NBT (50 μM), riboflavin (10 μM), and 0.1 mL of an enzyme extract. The activity of SOD was determined spectrophotometrically at 550 nm and expressed as unit min^−1^ mg protein^−1^.

Peroxidase activity was evaluated using the approach of Nakano and Asada [93]. The peroxidase activity reaction mixture consisted of phosphate buffer (100 mM, pH 7.8), guaïacol 20 mM, H_2_O_2_ 40 mM, and 0.1 mL of enzyme extract. Readings were measured at 470 nm and the results were expressed in μmol guaïacol mg^−1^ protein min^−1^.

For the calculation of PPO activity, catechol 20 mM in phosphate buffer (0.1 M, pH 7.8) and 0.1 mL of vegetal extract of the sample were used to make the reaction mixture. PPO activity was estimated at a wavelength of 420 nm and expressed in μmol mg^−1^ protein min^−1^ [94].

### 4.10. Plant Nutrient Uptake Analysis

After harvesting, the mineral nutrients (N, P, K) were measured in cactus cladodes from each treatment. The samples were oven-dried, then finely crushed before 1 g was added to digestion tubes and digested with H_2_SO_4_. The Kjeldhal technique was used to analyze the plant filtrate for nitrogen and the spectrophotometric method for phosphorus concentrations [95,96]. Flame photometry was used to measure cladodes’ potassium content [97].

### 4.11. Statistical Analysis

The averages of three replications (n = 3) were used to calculate all the results. ANOVA 1 SPSS 23 software was used for data analysis (Tukey test, *p* 0.05). A significance level of *p* ≤ 0.05 was used to determine the statistical significance of the treatment effects. A principal component analysis (PCA) was carried out using XLSTAT v. 2016 in order to examine the different interactions among the variables and the various treatments applied.

## 5. Conclusions

Conclusively, the current findings clearly show that the exposure of cacti to severe water stress over four months affects their performance. Moreover, Hs and/or AMF application mitigated the adverse effect of drought to a significant level.

In many ways, the advantages of applying Hs and/or AMF were obvious. They significantly reduced the drought-induced reduction in cactus growth, mainly by increasing the nutrient supply and absorption (N, P, K) and photosynthetic capacity, fortifying the antioxidant system (reducing stress markers and improving antioxidant activity), and fostering osmolyte accumulation.

Besides the impact on plants, Hs and/or AMF had a positive influence on soil quality through promoting soil aggregation, organic matter content, and nutrient levels, showing the potential of these biostimulants to boost soil health.

Research suggests that utilizing natural resources such as Hs and AMF can be an effective ecological approach to enhance the growth performance of cacti under drought, thus serving as a valuable tool for promoting plant growth under water scarcity.

## Figures and Tables

**Table 3:** Effects of humic substances (Hs) and/or arbuscular mycorrhizal fungi (AMF) on cladode mineral composition of *Opuntia ficus-indica* under various water regimes.

Treatments	Water Regime	Phosphorus (mg/g)	Nitrogen (mg/g)	Potassium (mg/g)
T	Without irrigation	11.44 ± 0.22 ^f^	3.93 ± 0.11 ^g^	11.02 ± 0.47 ^i^
	15% F.c	11.87 ± 0.25 ^f^	3.65 ± 0.20 ^g^	11.36 ± 0.24 ^h^
	30% F.c	18.88 ± 1.11 ^e^	6.71 ± 0.15 ^c^	15.25 ± 0.67 ^e^
Hs	Without irrigation	18.15 ± 1.54 ^e^	4.75 ± 0.19 ^fg^	14.56 ± 0.41 ^ef^
	15% F.c	21.67 ± 0.19 ^cd^	5.02 ± 0.24 ^e^	15.86 ± 0.68 ^e^
	30% F.c	22.08 ± 0.28 ^b^	7.98 ± 0.20 ^b^	21.35 ± 0.41 ^a^
AMF	Without irrigation	20.74 ± 0.31 ^d^	4.41 ± 0.23 ^g^	13.20 ± 0.72 ^g^
	15% F.c	24.44 ± 0.28 ^ab^	4.57 ± 0.43 ^f^	13.25 ± 0.81 ^g^
	30% F.c	26.81 ± 0.28 ^a^	7.68 ± 0.37 ^b^	18.36 ± 0.44 ^c^
Hs+AMF	Without irrigation	18.75 ± 0.46 ^e^	5.35 ± 0.37 ^e^	14.56 ± 0.39 ^ef^
	15% F.c	21.85 ± 0.22 ^c^	5.74 ± 0.31 ^de^	16.48 ± 0.57 ^d^
	30% F.c	23.94 ± 0.15 ^ab^	8.31 ± 0.57 ^a^	20.93 ± 0.35 ^b^

T: control plants; Hs: plants amended with humic substances; AMF: plants inoculated with AMF; Hs+AMF: plants amended and inoculated with Hs and AMF; F.c: field capacity. The data presented are the mean (±standard error) of three replicates and different letters show significant differences at *p* ≤ 0.05.

## Data Availability

Data are contained within the article.
